# P-180. Enhancing Mortality Surveillance: Insights from Excess Deaths Analysis during the COVID-19 Pandemic

**DOI:** 10.1093/ofid/ofae631.385

**Published:** 2025-01-29

**Authors:** Sinisa Skocibusic, Seila Cilovic Lagarija

**Affiliations:** Institute for Public Health of the Federation of Bosnia and Herzegovina, Mostar, Federation of Bosnia and Herzegovina, Bosnia and Herzegovina; Institute for Public Health of the Federation of Bosnia and Herzegovina, Mostar, Federation of Bosnia and Herzegovina, Bosnia and Herzegovina

## Abstract

**Background:**

The global repercussions of the COVID-19 pandemic extend beyond reported fatalities, imprinting a lasting effect on global health. The COVID-19 pandemic has presented substantial issues, encompassing not only direct infections but also excess mortality. The death rates in the Federation of Bosnia and Herzegovina (FB&H) have been significantly affected by the pandemic, leading to a need for a thorough analysis of the elements that contribute to this phenomenon.

Mortality in Federation of Bosnia and Herzegovina age 15 - 44

**Figure:**
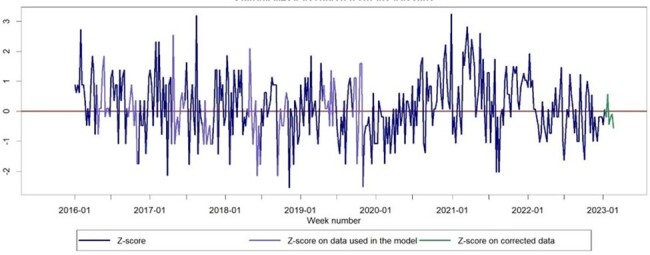

**Methods:**

The objective of this study was to quantitatively assess the rise in overall excess mortality rates in the FB&H region for the years 2020, 2021, and 2022. We employed data from the FB&H mortality record, along with population statistics and official COVID-19 death counts, to emphasize the often underestimated number of deaths. Our objective was to offer a thorough comprehension of the actual scale of the pandemic.

Mortality in Federation of Bosnia and Herzegovina age above 65

**Figure:**
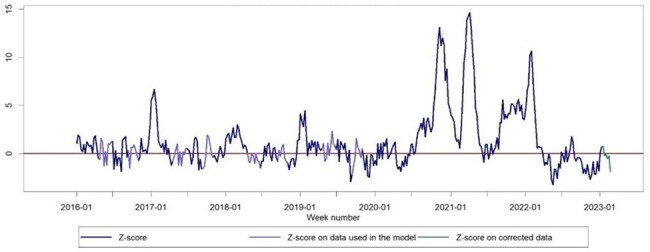

**Results:**

During the initial three years of the pandemic, FB&H encountered three separate instances of heightened mortality from any cause, accounting for 18 % of the overall excess mortality during the period of 2020–2022. The biggest burden was recorded among adults aged 45–74, with 22.4% falling within the 45–64 age group, 22.1% within the 65–74 age group, and 19.4% within the 75–84 age group. Children under 15 years old did not exhibit any excess mortality.

**Conclusion:**

COVID-19-related mortality in FB&H are significantly underreported, especially among younger persons. This statement highlights the pressing necessity to enhance civil registration and vital statistics, while pushing for the implementation of comprehensive surveillance systems for all causes of mortality. These measures are of utmost importance in improving the surveillance of forthcoming pandemics and effectively addressing other significant public health occurrences.

**Disclosures:**

**Sinisa Skocibusic**, Medicopharmacia: Travel grant to ECCMIDs

